# Acknowledgment to Reviewers of *Healthcare* in 2021

**DOI:** 10.3390/healthcare10020262

**Published:** 2022-01-29

**Authors:** Healthcare Editorial Office

**Affiliations:** MDPI AG, St. Alban-Anlage 66, 4052 Basel, Switzerland

Rigorous peer-reviews are the basis of high-quality academic publishing. Thanks to the great efforts of our reviewers, *Healthcare* was able to maintain its standards for the high quality of its published papers. Thanks to the contribution of our reviewers, in 2021, the median time to first decision was 20 days and the median time to publication was 43 days. The editors would like to extend their gratitude and recognition to the following reviewers for their precious time and dedication, regardless of whether the papers they reviewed were finally published:
Abdeldjalil OuahabiJolanta VaskelyteAbdel-Latif MohamedJon Anthony HyettAbdullah Al ShoyaibJon SchommerAbdullah WahbehJonas RanstamAbdulrahman AlwhaibiJonathan AveryAbel Bauero EscribanoJonathan D. KarpAbhirup DasJonathan DarrowAbhishek Kumar SinghJonathan KerrAbhishek SethJonathan N. FlakAbia Akebe Luther KingJonathan TritterAbigail ReiterJong Cheol ShinAbishek ChandrashekarJong-Hwa JangAbraham López VivancosJonhatan SilvaAchraf AmmarJoon Hyuk ChoiAda YanJooyoung KimAdam GoodworthJordi Tous PallarésAdam J. MilamJörg HaierAdam KernJorge Calvillo-ArbizuAdam LewisJorge Cerqueiro-PequeñoAdams BodomoJorge DazaAddisson SalazarJorge Esquiche LéonAdebowale OgunjirinJorge Góngora-RodríguezAdeboye AzeezJorge Humberto Palmeira RamosAdel AltiJorge Luis García-AlcarazAdela AndryskovaJorge Luís Victória BarbosaAdelaida AvinoJorge PérezAdélaïde BlavierJorge Pérez-GómezAdhikarimayum Lakhikumar SharmaJorge-Manuel DueñasAdilson MarguesJose A. BetancourtAdina Turcu-StiolicaJosé A. CernudaAdoración GraciaJosé A. F. CostaAdrian CurtoJosé Afonso NevesAdrian Elmi-TeranderJosé AlvarelhãoAdrian HarrisonJosé Angelo BarelaAdrian ŁukowskiJosé Antonio Ariza MontesAdriana AlexandruJose Antonio de PazAdriana HenriquesJosé Antonio MingoranceAdriano M. PimentaJosé Antonio Morales-GonzálezAdriano PiattelliJosé Antonio OrtegaAdrie VerhoevenJosé Antonio Ponce-BlandónAdrienne SalmJosé Carlos Dias RoucoAdrija HajraJose CarugnoAdwitiya KarJose Daniel Jimenez-GarciaAffan ShoukatJose De Albuquerque Calasans-MaiaAfona ChernetJosé Garcia-ArroyoAfonso M. CavacoJose Granero-MolinaÁgata AranhaJosé Hernández OrtegaAgata M. NiewczasJosé Ignacio Calvo ArenillasAgata SzulcJosé Javier Romero - Díaz De La GuardiaAgata TrzcionkaJosé Luis Gómez-UrquizaAglaja StirnJosé Luis Martín-ContyAgneta Malmgren FängeJose Luis Vazquez-AvilaAgnieszka KujawskaJose M. Férnandez-BataneroAgnieszka KulikJosé M. MestreAgnieszka LasotaJosé Manuel Granada-LópezAgnieszka Lecka-AmbroziakJosé Manuel MendesAgnieszka MicekJose Manuel RamirezAgnieszka MlynarskaJosé Manuel Saiz-ÁlvarezAgnieszka TruszkowskaJose María León-RubioAgnieszka WareńczakJosé María Marcos-MerinoAgustín Aibar-AlmazánJosé MendesAgustin Gonzalez-VicenteJosé Miguel Mansilla DomínguezAhmad JalalJosé Miguel Martínez SanzAhmad KantarJosé Miguel Seguí-RipollAhmed Jibril AbdiJosé Niño-MoraAhmet BaydurJosé Pedraza ChaverriAida BiancoJosé Pedro MartinhoAida PetcaJosé Pedro Ramos RequenaAide MaldonadoJose-Benito Rosales ChavezAiham QdaisatJosé-María León-RubioAimé Lay-EkuakilleJosé-María Sánchez-GonzálezAimen KhacharemJoseph BaumgardenAinhoa Fernández-AtutxaJoseph K. BelanoffAinhoa Ulibarri OchoaJoseph KvedarAinoa Roldán AliagaJoseph PlasekAirlane Pereira AlencarJosh JesselAisha SirajJoshua Porat-DahlerbruchAitor Oyarbide-ZubillagaJoshua Warren SappenfieldAjay PalJosip TomićAjay Pratap SinghJoyce ChangAkebe Luther King AbiaJoycelyn LeeAkebo YamakamiJozef SimenkoAkhil VaidJuan Antonio Párraga MontillaAkhila VeerubhotlaJuan Antonio Valera-CaleroAkihiro ShiinaJuan Bautista De SanctisAkiko MizunoJuan C. De Agustin-AsensioAkinori HigakiJuan Diego Ramos-PichardoAkira MonjiJuan Evaristo Callejas-JerónimoAkira ShibanumaJuan F. BlancoAkos DobayJuan Francisco Velarde-GarciaAkshara RaghavendraJuan González-MartínezAkshay KumarJuan Jesús García-IglesiasAkshaya BhagavathulaJuan José Gonzalez GerezAlaa DaudJuan José González-BadilloAlain MassartJuan Manuel Muriel-SánchezAlarico ArianiJuan Miguel Fernandez CampoyAlbert NienhausJuan Mozas-MorenoAlberto BalduzziJuan Pedro MartínezAlberto BeharJuan Pedro Martínez RamónAlberto BollettaJuan Rodríguez MansillaAlberto Comesaña-CamposJuan Sebastián Fernández-PradosAlberto Frutos Pérez-SurioJuan Uribe-TorilAlberto Gómez-MármolJudith Van Der WaerdenAlberto González-GarcíaJudyta JuranekAlberto MaccariJue LiuAlberto ModeneseJulia A. ScottAlberto Quílez-RobresJulia BujanAlberto SardellaJulian McDougallAlberto SardiJulie Aitken SchermerAldemir B. Oliveira-FilhoJulie BeneshAlejandra Alonso-CalveteJulie C. AbayomiAlejandra Garcia-AlonsoJulie RibesAlejandro CarrascoJulie WalabyekiAlejandro Gil-SalmerónJuliet L. KingAlejandro SanzJulio C. De La Torre-MonteroAlejandro SilvaJulio CostaAleksandar VišnjićJulius NukpezahAleksander Jerzy OwczarekJumin ParkAleksandra Karczmarska-WódzkaJun KohyamaAleksandra KristoJun MiyataAleksandra KrolikowskaJun SongAleksandra RogowskaJun YasudaAleksandra RyłJunbo WangAlessandra BergadanoJung KimAlessandra FiorettiJungsuk KangAlessandra M. FerraroJung-won KwonAlessandra PutrinoJunhong ParkAlessandra RenziniJunlei QiAlessandra VitanzaJunxiang ChenAlessandro AlaimoJuraj NemecAlessandro FeolaJurgita AntuchevičienėAlessandro MalobertiJustin MatusAlessandro ManconJustyna Opydo-SzymaczekAlessandro N. VargasJustyna RójAlessandro NotaJuyeun LeeAlessandro PirasK. H. Katie ChanAlessandro PorrovecchioKacper NijakowskiAlessandro SanturroKa-Fu YungAlessandro ScalaKai CaiAlessandro ScanoKai ZhouAlessandro SolipacaKai-Cheng WangAlessandro SteccoKaining ZhiAlessandro StefaniniKalaimani ElangoAlessandro ZerbiKan SaitoAlessandro ZorziKang Hao CheongAlessia SpadaKannan ThanikachalamAlessia TognettoKaori KitagawaAlessio ContiKara A. Gray-BurrowsAlessio FacchinKarel AllegaertAletta MillenKarel D. CapekAlex PagnozziKaren Sokal-GutierrezAlexander DomnichKarin ConinxAlexander E BerezinKarin D. MartinAlexander KeulKarin KirchgatterAlexander KirpichKarine PanicoAlexander RobitzschKarissa PeyerAlexander SchultzeKarl AndriessenAlexandra AlbuquerqueKarl GlocknerAlexandra CernianKarl HeleAlexandra Ibáñez EscribanoKarol DąbrowskiAlexandra Pérez-FerreirósKarol SteckiewiczAlexandre BarbosaKarolina KossakowskaAlexandre ReynaudKarolina KowalczykAlexei N. SuminKarolina KrysinskaAlexey MelnikovKarolina Szymaniec-MlickaAlexis L. MrazKarthik RagunathanAlfonso Alvarado-LorenzoKasper G LauridsenAlfonso InfanteKassiani TheodorakiAlfonso J. Gil-LopezKatalin PetőAlfonso LorenzoKatarzyna GrocholewiczAlfredo CaturanoKatarzyna JagielskaAlfredo CiniglioKatarzyna Kajak-SiemaszkoAlfredo G. CasanovaKatarzyna ŁubiechAli BoolaniKatarzyna Mocny-PachońskaAli FakihKatarzyna NabrdalikAli FarahaniKatarzyna PiotrowskaAli FarmaniKatarzyna PotyrałaAli KrztonKatarzyna Sienkiewicz-MałyjurekAli MobasheriKatarzyna SikorskaAli S.M. JawadKate CollieAlice MannocciKate StephenAlice NoblinKateřina IvanováAlicja DomagalaKatharina DiehlAlina CernasevKatherine A. BirchenallAlina SchartnerKatherine ChenAlissa M BanyaszKatherine Chiu Man LeungAllison HodgeKathleen J. FarkasAllison RosenzweigKathleen MonahanAllysson Allex AraújoKathryn Lynn LoveroAlok PaulKatielle SilvaAlvarado-Ibarra MarthaKatja BoehmÁlvaro Astasio-PicadoKatja PynnönenÁlvaro Briz-RedónKatri JalavaÁlvaro Francisco Lopes De SousaKavita SinghAlvaro Lopes DiasKavitha PalaniappanAlvin Lee DayKay-Dietrich WagnerAlyssa HuffKayleigh DenyerAlyssa K. McGonagleKazuhiko HashimotoAmad ZafarKazuhiro P. IzawaAmalia SilleroKazuki IdeAmanda MisselKazuya InokiAmanda RadulescuKeane WheelerAmar ParvateKedra WallaceAmaya Erro GarcésKei SaitoAmaya PratKeiko Ogawa-OchiaiAmbarish P. BhatKeisaku FujimotoAmber GarciaKeisuke IzumiAmbrogio P. LonderoKeita HiraiAmelia DíazKeita KouzuAmelia V. García-LuengoKeith FeldmanAmerigo GiudiceKeith MorrisAmira GuirguisKeith PecorAmira Mohammed AliKelly ByrneAmir-Houshang ShemiraniKelvin WallsAmirreza KhodadadianKelvin WongAmit AlamKemmyo SugiyamaAmit ChandelKen Ing Cherng OngAmlan HaqueKenan LiAmmar AltemimiKenji HayashidaAmr Abdulfattah Hausien HassanKenneth A. EarleAmr HassanKenneth A. EatonAmritlal MandalKensaku MatsunamiAmutha RamadasKenta FurutaniAmy IshamKerry MansellAna AdanKerstin HamannAna Bela Sarmento-RibeiroKerstin RohdeAna Belén Barragán MartínKeshrie NaidooAna Belén Fraile-BermúdezKeun Ho RyuAna Belen Tulcanaza PrietoKeun-Yeong JeongAna BorovečkiKe-Vin ChangAna C. PinhoKevin ChenAna Carla BatissocoKeya SenAna CerveraKeyla Vargas-RománAna CoelhoKhaled Elsayed AhmedAna Corte-RealKhalfalla AwedatAna Filipa SilvaKhattak NisarAna Gorini Da VeigaKhushminder RaiAna Isabel BorgesKi Jin JungAna Isabel Corregidor-SanchezKihong HongAna Jurinjak TusekKil-Soo KimAna Luísa RodriguesKim BlomqvistAna María Calderón De La BarcaKim DooleyAna María García-ArranzKimitoshi YagamiAna Maria Gonzáles RamosKingsley E. AghoAna Maria StanciucKiran SandhuAna Maria Vazqquez CasaresKirstie GalbraithAna Rita FariasKirstin VachAna Salomé PiresKishu RanjanAna SaniKitamura MineakiAna Solari NeumannKitsaras GeorgeAna ValenciaKiyoshi TakagiAnabela Correia MartinsKiyotaka UchiyamaAnahita Jablonski-MomeniKlara KomiciAna-Maria CazanKoch AndreasAnamaria SavuKohei UchimuraAnand Kumar MishraKohkichi MorimotoAnand SinghKoichi UemuraAnastasia SaadeKoichiro KawaguchiAnca SimionescuKoji HosomiAnderson Ribeiro DuarteKoji KanayamaAndoni Carrasco-UribarrenKoji OtaniAndré AraujoKoji YamatsuAndré FiebigKok Yong ChinAndré Neves BarreirosKon Hee KimAndré NovoKonosuke NakajiAndré P. WolffKonrad AumannAndré Pimenta FreireKonstantinos KafetsiosAndré RamalhoKonstantinos KatogiannisAndre RodackiKonstantinos KotsisAndrea AgugliaKonstantinos LiopyrisAndrea BonassiKonstantinos StergiosAndrea BrugnoloKornelia BatkoAndrea Cecilia Serge RodríguezKossakowska KarolinaAndrea CioffiKota KodamaAndrea Clemente PalmierKouichiro KawanoAndrea Dall'AstaKoyin ChangAndrea DemecoKrishna Prahlad MaremandaAndrea FabboKrishnan GanapathyAndrea FariniKrishnendu KhanAndrea GalosiKristijan BecicAndrea GuazziniKristina KuhbandnerAndrea Herrero-CerveraKristine GebbieAndrea KroekerKritika PoudelAndrea L. Huseth-ZoselKrutika PatelAndrea MasciadriKrystal LynchAndrea PompignaKrystyna PawlasAndrea PuglieseKrystyna PoznańskaAndrea RegnerKrzysztof JurekAndrea Roxanne J. AnasKrzysztof KaczmarekAndrea SalfingerKrzysztof KrystaAndrea SalustriKrzysztof LetachowiczAndrea SambriKrzysztof SztankeAndrea StoccoroKshama ShahAndrea VitaliKuldeep BansalAndreas BuettnerKun YangAndreas IhleKun-Lin TsaiAndreas LipphausKuo-Ching MeiAndreas NilssonKurt A. EscobarAndreas PesterKurt AmmerAndreas SeitzKurt SvardsuddAndree HartantoKusheng WuAndreea BrabeteKyle BurghardtAndrei RusuKyle RichAndrei SurguchovKyle Y. WhitfieldAndreja Trojner-BregarKylie BaileyAndrés A. Fernández-FuertesKyoko AsakuraAndrés AgudeloKyoung Shin ParkAndrés Da Silva CandalKyriaki KalimeriAndrés García-GómezKyriaki MyrissaAndrés Gené SampedroKyu Young ChoiAndres Iglesias PrietoKyulee ShinAndrés J. PrietoKyung Eun LeeAndrés Sánchez-PradaKyung-hun KimAndrew C. PickettKyung-Hyun SuhAndrew CollierKyung-Yong ChungAndrew E. LittmannKyuwan LeeAndrew LaneL Douglas RiedAndrew MayersLaith AbualigahAndrew P. LavenderLakesha AndersonAndrew P. McGrathLambert SpaanenburgAndrew SchumannLanette StuckeyAndrew SoundyLanlan (Lacey) ChuAndrew TubbsLapaire Jean-RémiAndrew W. JoyceLara KularAndrzej AdamskiLara McClureAndrzej CzamaraLars E. OlssonAndrzej J. PiotrowskiLars KayserAndrzej JanowskiLaura Andreu-PejóAndrzej PacanaLaura AtzoriAndrzej PolanczykLaura BergantiniAndrzej RaszkowskiLaura C. Sánchez-SánchezAngel Belzunegui-ErasoLaura De Armas-RilloAngel De JuanasLaura Del Carmen Sánchez SánchezAngel GentoLaura Delgado-LobeteÁngel Martínez-SánchezLaura FreemanAngel Romero ColladoLaura LaffranchiÁngel Romero MartínezLaura López-LópezAngela AifahLaura MihalacheAngela DaddowLaura Muñoz-BermejoÁngela María Ortega-GalánLaura NathansAngélica De AntonioLaura W WesseldijkAngeliki AngelidiLaureline BerthelotAngelo B. HookerLauren A. FowlerAngelo BellinviaLauren M. SmithÂngelo JesusLauren Miller-LewisAngelo RosaLaurence CheungAnh NguyenLaurencia GovenderAnibal CoronelLaurent BélecAnil Kumar JonnalagaddaLaurent DonzéAnja GeislerLaurent MetzingerAnja IvicaLaurent SuppanAnja Kathrin JaehneLaurie MarrauldAnja QuastLavinia MoleriuAnke Renate Maria KraftLawrence BurnsAnkit ManglaLea TimmermannAnkita ShuklaLeigh HaydenAnn Katrin KraeuterLeire AperribaiAnn SvenssonLenin PavonAnna Adamus-MatuszyńskaLeona GilbertAnna BartosiewiczLeonardo Alexandre Peyré-TartarugaAnna BednarekLeonardo Azael García GarcíaAnna BocchinoLeonardo Catalano IniestaAnna E. Van Der WathLeonardo CesanelliAnna EdgestonLeonardo Juan Ramirez LopezAnna Garus-PakowskaLeonardo RundoAnna KajdyLeonidas IoannouAnna LeśkówLeonie HalloAnna LipertLeonie ShortAnna MadraLeonzio FortunatoAnna Maria PaolettiLetizia AppolloniAnna MertasLiana PlesAnna PaczkowskaLiang HuAnna PodlasekLiang Ting LinAnna RemkováLida MademliAnna Rita BiliaLidia GavicAnna Rosa DonizzettiLien DosscheAnna Tyrańska-FobkeLifeng ZhouAnna ValenzanoLígia Isoni AuadAnna Zalewska-JanowskaLiliana RogozeaAnnalisa CarlucciLilly AugustineAnne DirksonLin QiuAnne FalcouLinbo ZhaoAnne FasslLinda DynanAnne-Christine PlankLinda F. MartinAnnemarie E. BennettLinda GoodfellowAnnika EklundLinda HagedornAnnsofie AdolfssonLingling MiaoAnqi ZhangLinyi WangAnroop NairLisa ChristAnshika JainLisa GrantAnshul SrivastavaLisa HaleAnthony D. AndreLisa Kouladjian O'DonnellAnthony HerbertLiu (Lydia) YangAnthony L. KomaroffLiudmila LiutskoAnthony OlsonLoick MenvielleAnthony SinLoknath ChenAntoine ChatrenetLong LiuAntoine DurrbachLora HromadikAntonella ArghittuLoredana BellantuonoAntonella FrassanitoLoredana Sabina Cornelia ManolescuAntonella LopezLorenz S. NeuwirthAntonia AzziniLorenzo GianquintieriAntonia MarsdenLorenzo PolimenoAntonia RussoLorenzo RusticoAntonija TadinLorenzo TonettiAntonino MorabitoLorenzo VigentiniAntonino NaroLoreta Buksnyte–MarmieneAntonio AmmendoliaLoris NanniAntonio AquinoLouie Mar A. GangcuangcoAntonio BaldassarreLouise HillerAntónio CaleiroLouise MobergAntonio CárdenasLouise UnderdahlAntonio CicchellaLuana Tenorio LopesAntonio Córdoba FernándezLuca ArdigòAntonio CorselloLuca BerteroAntonio CorteseLuca D. BertzbachAntonio Daniel García RojasLuca D'AcciAntonio Del CasaleLuca FalzoneAntonio Hernández FernándezLuca FlesiaAntonio Jesús Molina FernándezLuca FreddiAntonio Jesús Ramos-MorcilloLuca GallelliAntónio Manuel Neves VicenteLuca GiacomelliAntonio MarquesLuca Giovanni LocatelloAntonio MinopoliLuca PiemonteseAntonio MontañésLuca RollèAntónio NogueiraLuca RussoAntonio Palazón-BruLuca SimioneAntonio PercesepeLuca SpieziaAntonio Rivero-JuárezLuca Steardo Jr.Antonio Roberto ZamunerLuca TestarelliAntonio RomanoLucas DelmonicoAntonio Sarasa CabezueloLucia CastelliAntonio Sarria-SantameraLucia CorsiniAnuli NjokuLucia MangoneAnupam SinghLucia NatarelliAras BozkurtLucia TattoliAremis VillalobosLuciana LabancaAreti StavropoulouLuciano PironeAriadni SpyroglouLuciano RomanoAris BechlioulisLucie WaltersAriuntuul GaridkhuuLucy ArmitageArkadiusz JanuszewskiLudgleydson Ludgleydson Fernandes De AraujoArkadiusz StanulaLudo VanopdenboschArkaitz CastañedaLuigi BonavinaArkopaul SarkarLuigi CipolloniArmanda PereiraLuigi Di TommasoArmando AliuLuigi LoscoArmando CuttanoLuigi VetrugnoArmin KrausLuis Albendín-GarcíaArnab MukherjeaLuis Alberto Gaitán-CepedaArnaud PagesLuis Alberto Rodríguez-PicónArnulfo Ramos-JiménezLuis Andreu CaravacaÁrpád TósakiLuis Augusto Eijy NagaiArrigo CiceroLuís Carlos MatosArryn A. GuyLuis Ceballos-LaitaArsen S. MikaelyanLuis Cobos-PucArtan HoxhaLuis E. Anido RifónArtemissia-Phoebe NifliLuis Enrique Valdez-JuárezArthur De Sá FerreiraLuis Fernando MachadoArthur FerreiraLuis Gerardo de la FragaArturo Sanchez-PerezLuis Gómez PérezArye L. HillmanLuis Ignacio Lopera GonzálezAsbjørn ÅrøenLuis Llamas-AlonsoAsborg BjertnaesLuis López-FloresAscensión Palomares RuizLuis Manuel Fernández-CachoAsel RyskulovaLuis Manuel Mota De SousaAshkan Ghanbarzadeh-DagheyanLuis Miguel Cairós-VenturaAshwin AshokLuis MöckelAsrat AmnieLuis Naranjo-ZeledónAstrid‐Jane WilliamsLuis Parrilla RoureAthanasia PrintzaLuis Rodrigo MartínAthanasios KakarountasLuis SousaAthanasios TselebisLuis Suarez OgnioAthanasios ZervasLuís TinocaAtheer ZgairLuis VitettaAtsushi IrisawaLuisa Losada PuenteAttà NegriLuiz CarneiroAttayeb MohsenLuiz Fernando Romanholo FerreiraAttila FrankoLuk Hsiang NingAttila FrigyLukai LiuAtul Kumar PandeyLukas RasulićAugustine KangŁukasz BalwickiAurel Popa-WagnerŁukasz PałkaAurelio ArnedilloLuke BalcombeAviad Tur-SinaiLuke WoonAvinash AujayebLung ChanAxel SteigerLuuk Van KempenAya FujiwaraLuxana Reynaga-OrnelasAykut TamerLynissa StokesAzadeh NilghazLynn LevatteAzam AslaniLynnie CorrellB. JanetM. Angeles BeleñaBabatunde RajiM. Carmen Terol CanteroBadar Nadeem AshrafM. Gloria Gallego-JiménezBalan RathakrishnanM Idoia Ugarte GurrutxagaBarbara AndersonM. Jean KellerBárbara AngelMaarten Van CleeffBárbara Badanta RomeroMaayan ShachamBarbara BaranowskaMaciej FrantBarbara BezerraMaciej GawęckiBarbara Bodner-AdlerMaciej Iek SkrzypczakBarbara CviklMack ShelleyBarbara ŁapińskaMaddalena NapolitanoBarbara MazurMadeline SprajcerBarbara NazareMadelon L. FinkelBarbara PieperMadepalli LakshmanaBarbara RuaroMadison SilversteinBaseer U. AhmadMael LeverBayodé Roméo AdégbitèMaestri EnricoBeadaa Jasem MohammedMagda GioiaBeata BucheltMagdalena A. BerkowskaBeata JabłońskaMagdalena Martínez-CañameroBeata SzluzMagdalena SkarzynskaBeata Zyznarska-DworczakMagdalena WrzesińskaBeatriz GarcíaMagnolia CardonaBeatriz Gómez-MartínMahbubul ShihanBeatriz Núñez AnguloMaheswari MuruganandamBee Kang TanMahmood YousefiBefikadu WubishetMahmoud ElbattahBegoña EspejoMai Salah El Din Mohamed HelmyBegoña GuiraoMaik BielekeBehnam GhasemzadehMaite E. Rivera GorrínBelem BarbosaMaja WrzesienBenan BayrakciMajed OdehBénédicte M. BatrancourtMajid KoozehchianBengt KayserMaju KuriakoseBenito León del BarcoMakayla CordozaBenjamin AnsaMaksymilian GajdaBenjamin CullMalcolm KooBerna RahiMałgorzata BronikowskaBernhard RauchMałgorzata ChlabiczBernhard WernlyMałgorzata DołowyBersain A. ReyesMalgorzata Gamian-WilkBertrand DautzenbergMałgorzata JamkaBethesda O'ConnellMałgorzata LehnerBetsy Weening-DijksterhuisMałgorzata Lesińska-SawickaBetty DoreMalgorzata MaciukiewiczBeverly HoganMalgorzata PankowskaBhavana PalakurthiMałgorzata StachońBhavuk GargMałgorzata StefaniakBhupal BanMalik SallamBiagio RaponeMalik YousefBibhuti K. SarMalin Lohela-KarlssonBidisha RoyMallikharjun KoriviBill JesdaleMallory MarshallBin ChenManela GlarcherBin DuManfred BornewasserBinh Q. TranManoj SharmaBirgit BabitschManuel Albornoz-CabelloBjörn WeissManuel Arias-MontielBlanca De-la-Cruz-TorresManuel Au-Yong-OliveiraBlanka StiburkovaManuel Casal-GuisandeBlas Lotina-HennsenManuel Castro-SanchezBo Hyun ParkManuel Contreras-LópezBo PangManuel Durán PovedaBogdan SoceaManuel Florindo Alves MeirinhosBoguslawa UrbaniakManuel Franco-MartínBoleslaw KarwowskiManuel GrañaBonhyuk GooManuel Isidoro CapelBooth Catherine MakisonManuel Monfort-PañegoBoris De RuyterManuel Pabón-CarrascoBorja Hernández-BreijoManuel PereaBorja Herrero De La ParteManuel PereiraBorja Matías PompaManuel Pestana VasconcelosBoro ŠtrumbeljManuel Prado-VelascoBor-Shiunn LeeManuel Rodríguez-HuguetBo-Wei ZhuManuela Expósito-RuizBrach PostonManuela SilvaBrad StennerMarc JacquinetBraden BagleyMarc LochbaumBranden NemecekMarc PilkingtonBrandon GaudianoMarc Woodbury-SmithBrandon Lucke-WoldMarcello MaddaloneBranka StojanovicMarcello MocciaBrent PetersonMarcelo GitiranaBrett VaughanMárcia BarrosBrian C. J. MooreMarcin ButlewskiBrian K. FoutchMarcin CzechBrian OndiegeMarcin JozwikBritt ÖstlundMarcin RzeszutekBrodie FraserMarcin SochalBruce Baer ArnoldMárcio De Carvalho FormigaBruce J. BarronMárcio OliveiraBruce McLucasMarco Antonio Cruz-MoratoBruce SunderlandMarco BiagiBruna BrunoMarco CascellaBruno BalbiMarco ClariBruno Bueno-SilvaMarco FarronatoBruno ChrcanovicMarco FonzoBruno José Nievas SorianoMarco Matteo CicconeBruno MagalhãesMarco MeleBruno Miguel VelosoMarco MiglioratiBugra AlkanMarco RavinaBülent ErenMarco RoccettiByoung Sun ChuMarco RocchiByoung-Hee LeeMarco RosinaByung Yong JeongMarco RuggieroByung-Jik KimMarco TjioeByung-Sun LeeMarco Trabucco AurilioCalogero Lo DestroMarco ZamoraCamelia SoponaruMarcos José RenniCamilla PasternackMarcus LancéCarey MatherMarcus SchiltenwolfCarla Maria Batista Ferreira PiresMarek GancarzCarla SerrãoMarek KieliszekCarla Sofia SilvaMarek KrzystanekCarles BarriocanalMarek LapkaCarlo CampanaMaren GoeckenjanCarlo GalliMaren WeissCarlo Giacomo LeoMargaret Eleanor CruickshankCarlo LaiMargaret FitchCarlo LajoloMargaret K. YuCarlo PerriconeMargaret RedelmanCarlos AbreuMargaret SouzaCarlos Dias MacielMargareta LiljaCarlos GonçalvesMargarida VieiraCarlos LaranjeiraMargarita Pino-JusteCarlos Llanes-ÁlvarezMargherita GiannoniCarlos López-de-CelisMargherita Micheletti CremascoCarlos López-GutiérrezMargherita ZitoCarlos Méndez-MartínezMargit SerbanCarlos Miguel MartoMargot WM De WaalCarlos Peñarrubia-LozanoMari VállezCarlos Ruiz-NuñezMaria Adele GiamberardinoCarlos S. Cruz-FuentesMaria Antonella De DonnoCarlos SalaveraMaría Antonia Parra-RizoCarlos SerraoMaria António CastroCarlos TrenadoMaria Aparecida Neves JardiniCarlos ValderramaMaria ArmaouCarlos VasconcelosMaría Arregui GambúsCarlos Vaz De CarvalhoMaria Assunta ZoccoCarlos Vladimir Zambrano RodríguezMaría Carmen RicoyCarme SaurinaMaría Cristina OrtizCarmen CasanovasMaria C.T. CangussúCarmen De KockMaria D’AccoltiCarmen Enrique-MirónMaria De Los Ángeles Cardero-DuránCarmen F. Navarro-PérezMaría Del Carmen Olmos GómezCarmen Moret-TatayMaría Del Carmen Rodríguez GarcíaCarmen Pérez-RodrigoMaría Del Carmen Valls MartínezCarmen RequenaMaría Del Mar Ferradás CanedoCarmen Romero-LópezMaría del Mar Jiménez LasserrotteCarmen Vizoso-GómezMaria Del Mar Sanchez-PerezCarmine FinelliMaría del Pérez-FuentesCarmine IzzoMaría De-la-Casa-AlmeidaCarmine ViolaMaría Dolores López FrancoCarol BashfordMaría Dolores Ruiz FernándezCarol LinMaria Elvira CorreaCarolina Alonso MonteroMaría Evarista ArellanoCashen M. BoccioMaria Giuliana VannucchiCatarina XavierMaria GrajdianCate ThomasMaria Grazia Lourdes MonacoCaterina GozzoliMaria Isabel MármolCaterina TramontiMaría Jesús Núñez-IglesiasCatherine EastonMaria João VelezCatherine GammonMaría José García-PolaCatherine Plüss-SuardMaria José VaradinovCayetano Medina-MolinaMaria KrogsethCecilia AriciMaría Lidón Moliner MiravetCecilie VarsiMaría Luisa Delgado-LosadaCédrick T. BonnetMaria Luisa IndianaCelia HildebrandMaria Luisa MarvánCésar Calvo-LoboMaría M. Morales Suárez-VarelaCésar CaraballoMaria Magdalena Bujnowska-FedakCésar CárdenasMaria MaiaCésar García-HernándezMaria ManconiCesare CerriMaria Mendoza-MuñozCezar HonceriuMaria Miklasińska-MajdanikChad SealesMaria Montiel TroyaChae Yeon ParkMaria Morgan-BathkeChae-gil LimMaría N. Moreno GarcíaChandra Sekhar BathulaMaría Nieves Pérez MarfilChandra SinghMaria PaparoupaChang Gue SonMaría Patrocinio Ariza VegaChang Won LeeMaria PieriChangqing LuoMaría Pilar Martinez-JimenezChangSeob YeoMaría Pilar MatudChao ChenMaría Pilar Pecci-LloretCharalampos SiristatidisMaria Rita BiancoCharat ThongprayoonMaría Rosa Magallón BotayaCharles AtkinsMaria Salem IbrahimCharles G. MinardMaría Salvadora Ramírez JiménezCharles IkerionwuMaría Sánchez-ZafraCharles MadewellMaría Soledad Prats MoyaCharli ErikssonMaria Teresa GuagnanoCharlotte VilliersMaría Teresa Iglesias LópezCharlotte Wendelboe-NelsonMaría-Arántzazu Ruescas-NicolauChee-Jan ChangMaría-Dolores Lanzarote-FernándezChelsea PelletierMaría-Leticia Meseguer-SantamaríaChen LingMarialisa ScatáCheng WangMarian BotchwayCheng-Fang YenMarian WoźniakCheng-Maw HoMarianne SilvaChen-Li LinMariano CingolaniChen-Long WuMariantonietta FrancavillaChen-Yi SongMariarita BrancaccioChenyu SunMarie Magdaleen BismarkCheong K ParkMarie-France Champoux-LarssonCheuk Lun LiuMarieke H.J. van den Beuken-van EverdingenChhaya PatelMarija LjubicicChiara BenedettoMarika MassaroChiara MarracciniMarilza Vieira Cunha RudgeChiara OppiMarin SenilaChiau Ming LongMarina AgozzinoChien-Feng LiMarina Amorim SousaChien-Hung LeeMarina ButovskayaChien‑Wen ChenMarina QuartuChi-kin LawMarina Sánchez-RicoChin Yi FangMarina SantaellaChin-Shiuh ShiehMarina SartiniChintalapudi NaliniMarino VilovicChirag AgarwalMario Alvarado-LorenzoChiranjibi SitaulaMario Bernardo-FilhoChong WentongMario BonominiChoon Guan LimMario Del Libano MirallesChoong Hyun KimMario Di TragliaChris GroendykeMario García-SuárezChris PaciMario José Costa De MacedoChristian CavéMario Lozano-LozanoChristian JanousekMario MiniatiChristian KrägelohMario VersaciChristian MesengeMário W. L. MoreiraChristian NapoliMariola DrozdChristian TanislavMariola PawlaczykChristian-François RoquesMarion RussellChristina LaspaMarion SlackChristine KeipertMarisol RodríguezChristine Manlai KwanMarius ColinChristine St LaurentMariusz DuplagaChristoph BorzikowskyMariusz GoniewiczChristoph LeonhardMariusz WysokinskiChristoph N. SeubertMariya KovalevaChristopher BallmannMariyana SchoultzChristopher BoehlkeMarjeta MaroltChristopher KobierzyckiMark HenricksonChristopher L. AtkinsonMark MeredithChristopher LambMark R. JohnsonChristopher LoMark ReybrouckChristopher MansourMark ScherChristopher MayerMark Ulrich GerbershagenChristopher OwensMark WillemsChristopher R. SnellMarko MarhlChristopher T. OwensMarkus PuchingerChristopher WilkinsonMarlies GijsChristos MalliarakisMarnie DobsonChristos MikropoulosMarny FedrigoChuan-Zhi DongMaroof AlamChun-An ChengMarquisette Glass LewisChun-Chieh KaoMarta Evelia Aparicio GarcíaChungho LauMarta MuszalikChung-Ho SuMarta RusekChung-Ying LinMarta TremoladaChun-Hung ChangMarta Wysocka-MincewiczChuong NgoMartha Patricia Gallegos-ArreolaChu-Shiu LiMartin BeerCid André Fidelis De Paula GomesMartin DaumillerCindy CrawfordMartin MaguireClara SimãesMartin Sanchez-GomezClara TangMartin TropeClaudia CurciMartin WettsteinClaudia DellaviaMartina FaraldiClaudia Iveth Astudillo-GarcíaMartine BaronsClaudia KitzmüllerMarwan El MobadderCláudia Maria Ferreira PereiraMarwan HabibaClaudia MehedintuMary DavisClaudia PisanuMary HiddeClaudia RusMary KamienskiClaudimar Pereira Da VeigaMary M.Y. WAYEClaudine FabreMary V. SeemanClement LorkMaryam ArdalanClementine LabroscianoMarzi ChiaraClifford GoodmanMarzulli MicheleClyde F. MartinMasa SasagawaColleen M GallagherMasaharu KagawaConcepción De-Hita-CantalejoMasahiro KitamuraConcetta PirontiMasahiro SeikeConsolación Gómez-ÍñiguezMasashi KanaiConstantino BalestraMasato KakuConstantinos KoutsojannisMasayuki OkudaConsuelo ChacarteguMasrour MakaremiConsuelo Gutiérrez-OrtízMassimiliano CretaConxita MestresMassimiliano MannoCordelia Estevez-CasellasMassimiliano PanellaCorey SellandMassimo CarossaCorina IoanășMassimo CrescimbeneCorina S. PopMassimo StellaCorinna CullerMassimo TusconiCorrado Lo StortoMassinissa YahiaCoskun Joe DizmenMat DalgleishCostas S ConstantinouMateusz GliwińskiCraig H. KennedyMateusz JankowskiCraig SinclairMateusz KuklaCristi SpulbarMateusz PusleckiCristián A. AmadorMatheus Henrique Dal Molin RibeiroCristian LieneckMatheus MarconCristian MartinMathews VargheseCristián OyanadelMathias ForjanCristian TimmermannMathilde ForestierCristiano AmarelliMatias NollCristina BaixinhoMatt RickardCristina CortisMatteo BolcatoCristina FrangeMatteo ChiappediCristina GalvanMatteo CrippaCristina García-AelMatteo InnocentiCristina García-BravoMatteo PaciniCristina Liébana-PresaMatteo PaganiniCristina M. Beltrán ArocaMatteo RiccòCristina MarognaMatteo RipaCristina MonteiroMatteo RitrovatoCristina Rivera-PicónMatthew DonaduCristina Roldán-JiménezMatthew WichrowskiCristina-Mihaela LăcătușuMatthias GatzCristoforo PomaraMatthias PairederCuauhtemoc Lopez-MartinMatthias ZeilerCynthia BlantonMattias PaulssonCynthia E. MuñozMattsson JanetCyrus MotamedMaura MacPheeDaiana Priscila Rodrigues-de-SouzaMaura PozziDaiva RastenytėMaureen RyanDamian C. HineMaurice MarsDamian HudziakMaurizio D’AmarioDamián LópezMaurizio SchiavonDamiana ScuteriMauro CeccantiDan VoiculescuMauro GiuffrèDana AlonzoMauro LizotDana CopotMauro MartinelliDani TostMauro RomanelliDaniel Cabanillas-BalseraMax MenziesDaniel CameronMaxime BourgainDaniel Gabaldón-EstevanMay Ee PngDaniel GonzálezMaya CormanDaniel KopaskerMeenakshi AhluwaliaDaniel LindsayMegan PennoDaniel López-PlazaMehmet AktasDaniel P. PotaczekMehmet GülDaniel SchulzeMei Shan LIEWDaniel Sobrido-CameánMei-Chi HsuDaniel TerryMei-Fang WuDaniela Acquadro MaranMeiyi MaDaniela CancelliereMelanie Reuter-OppermannDaniela DamianiMeliha SalahuddinDaniela DibelloMeline KevorkianDaniela FotiMelissa Agsalda-GarciaDaniela HaluzaMelissa CareyDaniela IacovielloMelissa N. SlavinDaniela OehringMeng-Che TsaiDaniela PierannunzioMenico RizziDaniele GiansantiMercedes Díaz-RodriguezDaniele SpoladoreMercedes Rizo BaezaDanielle GreenbergMervyn Márquez GómezDanielle Laure Taneyo SaaM.F. AhamedDanielle R. EvangelistaMi Ran EomDanijela Ristić-MedićMichael A. JacksonDanilo Medeiros ElerMichael BermanDanúbia Da Cunha De Sá-CaputoMichael ConnollyDanuta GajewskaMichael CopeDaria AugustyniakMichael D. FreemanDaria BylievaMichael EnwereDario BacchiniMichael EvansDario BruzzeseMichael JacksonDario ManfellottoMichael John NortonDario MessenioMichael LiebmanDario Piombino MascaliMichael MahanDariusz TworzydłoMichael MunzDarko AntičevićMichael O OgundeleDarko ModunMichael SteinbornDartagnan Pinto GuedesMichael SwashDaryl JonesMichael T. KoltzDaryl Wayne NiedermoserMichael TozeDave WheelerMichael UlloDavid Ahmedt-AristizabalMichael VannierDavid Alves Ferreira ÉvoraMichał BronikowskiDavid AparisiMichal CzaplaDavid Baeza MoyanoMichal JurášekDavid BentleyMichał RabijewskiDavid ColecchiaMichal S. NowakDavid Conde-CaballeroMichel JancloesDavid CromptonMichel LadouceurDavid Escudero-ManceboMichel PérusseDavid Hernández HerreroMichela RossiDavid J WashburnMichele AvanzoDavid JaspanMichele CassettaDavid KomatsuMichele CompagnoDavid L. BankesMichele D'AttilioDavid L. MayorMichele SabatinoDavid LucasMIchele SantoniDavid MaloneMichele TonelliDavid MoleroMichelle FloodDavid NalboneMichelle PrasuhnDavid Pérez JorgeMichelle Weber RawlinsDavid PerpetuiniMichio HongoDavid Sánchez-PorrasMiglė BacevičienėDavid Sancho-CantúsMiguel A. Montero-AlonsoDavid SchultzMiguel AlcarazDavid SchumacherMiguel Angel Alcántara-OrtigozaDavid SkvarcMiguel Ángel Echarte FernándezDavid StepenskyMiguel Ángel Gallard-VigilDavid Varillas-DelgadoMiguel Ángel Navas-MartínDavid Z. RoseMiguel Ángel PulidoDavide BarbieriMiguel Ángel Rojo TiradoDavide BorroniMiguel Castelo-Branco SousaDavide CalandraMiguel RicouDavide CavagnettoMiguel TerrosoDavide FerorelliMiguel TorresDavide Giuseppe RibaldoneMihaela ZegreanDavide Settembre BlundoMihajlo (Michael) JakovljevicDavide SistiMihir ShettyDawid KoźleniaMihnea-Alexandru GamanDawid PolapMikalai BudzevichDawid SigorskiMikhail F. BorisenkovDawn StobbartMikhail Yegorovich SinelnikovDe Gaulle I ChigbuMikio KajiharaDebasis BandyopadhyayMiklos BitayDeborah Alperovitch-NajensonMila A EmeraldDeborah J. BowenMilena LopreiteDeborah R. McFarlaneMin Sun KimDebra StroineyMina SamukawaDeepa AlexMinerva CruzDeepak KotiyaMing-Ju TsaiDeepanshu JainMing-Yi ShenDelicia Shu Qin OoiMinh Hoang NguyenDelvon T. MattinglyMinjeong AnDelyth Higman JamesMin-Seong HaDenis BourgeoisMircea-Catalin FortofoiuDenis KorneevMiriam Romero-LópezDenise HaslwanterMiriam Sánchez-SanSegundoDennis NiebelMirjam Van TilborgDerek F. H. PhebyMirka SaarelaDerek P. EvansMirko DuradoniDervla KellyMiro JukićDesiderio Cano PorrasMitch ClarkDhaval ChauhanMoacir MarocoloDhruvajyoti RoyMoawia KhatatbehDi JinMohab M. IbrahimDi ZhangMohamed Sameer AbdallahDiana E ValeroMohammad AlamDiana HeimesMohammad Nazmul HasanDiana Jiménez-RodríguezMohammad Rostami-NejadDiana-Alexandra ErtlMohammad SaadDiego Gomez-BayaMohammad ZamaniDiego Moreno-BlancoMohammed BindakhilDiego Sánchez-GonzálezMohammed S SalahudeenDiego Soto GarcíaMohannad W. KafriDiego Tlapa-MendozaMohd Parvez KhanDierdra BycuraMohsen MousaviDillon ChrimesMohsen SoltanifarDimitri PatrikiMoin U. AtiqueDimitrios KatsavelisMojtaba KhanesarDimitrios KorentzelosMojtaba VaismoradiDimitrios PanagopoulosMomcilo JankovicDimitrios VlachopoulosMona VintilaDimitris KarampatzakisMonica LöfvanderDimitris ZavrasMonica MoneaDina AveryMónica Ríos SilvaDina Di GiacomoMónica TeixeiraDinkantha KumararatneMonica TrifDiogo MendesMonika Dmitrzak-WęglarzDiogo MoraisMonika KlimekDmitriy V MazurovMonika ParchomiukDmitry GrigoryevMonika PiątkowskaDolores Rando-CuetoMonika SobočanDomingo J. Ramos-CampoMonokesh Kumer SenDominic HarmonMontserrat Gómez-de-Terreros-GuardiolaDominik GroßMonzurul RoniDominik RothMoosa PatelDomiziano TarantinoMoreno BaruffiniDonato LiloiaMorgane BaronDong-Hee RyuMoudatsou MariaDong-Her ShihMudasir A. KhandayDong-Kyu KimMuddassar SarfrazDonna M. PlattMudit TyagiDora SalesMuhammad Altaf KhanDoris KoflerMuhammad AsimDorota Bielinska-WazMuhammad Fazal IjazDorota ZyśkoMuhammad Hassan KhanDoru DiculescuMuhammad KamalDouglas McHughMuhammad Shahid IqbalDouglas SteinkeMuhammad Sheikh SadiDoyeon WonMuhammet KarakavukDragan KomljenovicMulazim Hussain AsimDragana KomnenovMunjae LeeDrucilla J. RobertsMuthu PeriasamyDu-Jin ParkMykola MakhortykhDulce MinayaMylène DuivonDuncan HillMyung Bae ParkDuo Wai Chi WongNadia CattaneDylan M. JohnsonNadia NajafiEce BayramNagisa SugayaEckhard FrickNa-hyeon (Hannah) KoEdit XhajankaNaiara OzamizEdith ClarosNaira DelgadoEdmanuel CruzNaji KharoufEdna PatatanianNakhle S. SabaEdoardo BraunerNancy HaskellEduard Moreno-GabrielNancy HelouEduardo A. F. NilsonNancy S. BolousEduardo Arroyo PardoNante NicolaEduardo Casilari PérezNaoki SudoEduardo OsunaNaoko ArakawaEdvard JohanssonNarayan RamamurthyEdward P. HofferNarendar DudhipalaEdward TolhurstNarrendar RaviChandranEdyta CharzyńskaNasreen LalaniEeva HarjuNassr Al-NuaimiEfrat NeterNatalia Alabaldejo BlázquezEfthimios DeligeoroglouNatalia Cezon-SerranoEhsan ArdjmandNatalia DowgiałłoEhsan MohammadiNatalia KucielEiichi SatoNatalie EnninghorstEiichi WatanabeNatalie MichaelsEileen CreelNatalya A. ShnayderEitaro KodaniNatalya KizilovaEladio Collado-BoiraNathalie AndréElaina Charlotte TaylorNathan K. EvansonElaine NguyenNathanael OjongElaine SouzaNathaniel GeyerElba MaurizNaushad Ali JungleeEleanor SteinNavchetan AwasthiEleanor WilsonNaveen KumarEleazar Aguirre AnayaNawab Muhammad Faseeh QureshiElena CancianiNazam DzolkarnainiElena Constanta AmăricăiNazareth Martínez-HerediaElena D BazhanovaNeda Haj-HosseiniElena GoriNeeraj BhandariElena GrossiniNeil GlossopElena Jiménez-PérezNekane SainzElena Labajo GonzálezNelli LyyraElena Martinez-SanzNelson A. De La Pena AtehortuaElena Ortega CamposNelson Andrade-GonzálezElena PiñeroNemany Abdelhamid Nemany HanafyElena PomariNemesio Villa-RuanoElena PrietoNeomi Vin-RavivElena RezusNeves-Amado JoãoElena TarcaNguyen Minh DucElena UrrestarazuNguyen Quoc Khanh LeElena ZaitsevaNicholas BelloEleonora NuceraNicholas T. BelloEleonora RicciNicola BaldiniEli D EhrenpreisNicola De AngelisElia Fernández-MartínezNicola LambertiElia RanzatoNicola MagnavitaElias MpofuNicola MarottaElikanah Olusayo AdegokeNicola MontemurroElin KjelleNicola NanteElio BorgonoviNicolas BergerElisa BelluzziNicolas PaulElisa CairraoNicole Cieri-HutchersonElisa ZavattaroNicole LevitzElisabet Roca-MillanNicole MarshElisabete BorgesNicoletta VegniElisabete P. SilvaNicolette Teufel-ShoneElisabetta BertelliniNidhi KapoorElisabetta NardellaNiels QvistElizabeth A. ReganNigel D RobbElizabeth C. PenickNihar KinarivalaElizabeth G. HunterNikesha J. GilmoreElizabeth KukielkaNikita GabdullinElizabeth OcholaNikola KomlenacElizabeth OnyangoNikolaos A. ChrysanthakopoulosElizabeth ParamythiotouNikolaos MachairasEllen FunkhouserNikolaos VassosElliot BenjaminNikoleta PrintzaElliot LassonNikolina Jukić PeladićEloy Lopez MenesesNikos ViazisElpidio Maria GarzilloNina LansburyElsa López-PintorNina ShinElsa PegadoNina TumosaÉlvio GouveiaNing QuÉlvio Rúbio GouveiaNing YuElvira Molina-FernándezNirit GavishElżbieta SzymańskaNirnath SahElżbieta WłodarczykNishel Mohan ShahEmanuele BarabinoNittari GiulioEmanuele BissoNobutaka KuriharaEmanuele D'AmicoNoelia María Martín EspinosaEmanuele MarzettiNoemí López-EjedaEmilia Inmaculada De la Fuente-SolanaNora Suleiman-MartosEmilie CameronNorbert GrafEmilien JeannotNorbert TripoltEmily HaasNorbert ZolekEmily RobertsNoriaki MaedaEmma BertucciNorihiko KikuchiEmma EspinosaNorio YamamotoEmma MotricoNoriyuki MatsukawaEmmanuel J FavaloroNorman David GoldstuckEmmanuel RineauNorzagaray Campos MarianoEng Tat AngNowicki AndrzejEnno van der VeldeNuno CrespoEnrico AmmiratiNuno GarridoEnrico BergamaschiNuno MoraisEnrico Fuini PugginaNuria FarreEnrico Mario CamporesiNuria Padrós-FloresEnrique MergelsbergOdette ChagouryEnza SperanzaOihana AristondoEric BravermanOke GerkeEric Francelino AndradeOlaf Chresten JensenEric P BorrelliOlatunde AremuErick Andres Perez AldayOlga GolubnitschajaErick Martínez HerreraOlga Maria López EntrambasaguasErik R. RanschaertOlga RedinaÉrika B. RangelOliver SanderErika Estrada CamarenaOllie Yiru YuErin McKinleyOlmo ZavalaErnest TyburskiOlumuyiwa James PeterErshuai ZhangOmar AlibrahimEsa Ala-RuonaOmar CauliEsperanza Begoña García NavarroÖmer ElmaEsteban Obrero-GaitánÖmer Muhammet SoysalEstela Calatayud SanzOrazio AielloEsther Cuadrado SotoOriol AmatEsther García-SánchezOrsolya NémethEsther LázaroOrtal SlobodinEsther MaasOscar CampuzanoEsther Medrano-SánchezOscar GarcíaEufrasio Pérez NavíoÓscar Gavín ChocanoEugene Y.H. YeungOsnat BashkinEugenia VlachouOthon PapadopoulosEun Jeong HwangOttar NessEunice CarrilhoOttmar HerchenröderEunjung RyuOtto LamEva Aguaded-RamírezOvidiu Popa-VeleaEva Ekvall HanssonPablo AquevequeEva León ZarceñoPablo Galvez RuizEva María Martínez-JiménezPablo Javier Olabe SánchezEva Warensjö LemmingPablo José Olivares-OlivaresEvan CleavePablo Monteagudo ChinerEvan RobertsPablo Olivares-OlivaresEvangelia ChrysikouPablo RuisotoEvelyne GozalPablo Valdés-BadillaEverton Falcão De OliveiraPablo Villalobos DintransEwa BaumPa-Chun WangEwa RzońcaPadulo JohnnyEwa SkarżyńskaPalina PrysmakovaEwa SobolewskaPaloma MassóF. Alethea MartíPamela TozzoF. Javier Del RíoPan FuminFabian HuettigPanagiotis IoannidisFabián Martínez HernándezPanagiotis K. MarhavilasFabiane Valentini Francisqueti-FerronPanagiotis KourtesisFabiano FerrariPankaj GaurFábio BitencourtPaola PiomboniFabio FabbianPaolo Davide FarahFábio FernandesPaolo DebertolisFabiola SilvaggiPaolo Emilio SantoroFabiola SpolaorPaolo Francesco ManiconeFabrizia GiannottaPaolo LauriolaFabrizio ConsortiPaolo MartellettiFady MansourPaolo SeverinoFahimeh Alsadat ShojaeiParisa GazeraniFaisal F. HakeemParmenion P. TsitsopoulosFalko FendParmenion TsitsopoulosFang TongParmit SinghFani GkrozouParul VarmaFarnaz FaridPasquale LongobardiFarnaz NickpourPat ThaneFátima FradePatricia AnafiFatima Leon LariosPatrícia BatistaFátima RoquePatricia Concheiro-MoscosoFatima SalahdinePatricia DiogoFatine EzbakhePatricia FennellFatmawati FatmawatiPatricia Grace-FarfagliaFaustin Armel Etindele SossoPatricia LoganFausto AssandriPatricia QuesadoFederica Di SpiritoPatricia Rocamora-PerezFederica FogacciPatrícia SoaresFederica GalliPatricia Takako EndoFederico RoggioPatricia VermeerschFeilong WangPatrick PangFelipa De Mello-SampayoPatrick WeydtFelipa SampayoPatrizia FarruggiaFelipe J. AidarPatrizia FerroniFelipe José AidarPatrizia GrifoniFelipe León MorillasPau Redón LurbeFelipe Ojeda PérezPaul B. KaplowitzFélix Marcos TejedorPaul BatchelorFeng YuanPaul E. EngelhardtFeng-Hua SunPaul Eric DossouFengyu LiPaul HibbardFerdinando AgrestaPaul KrausFERDINANDO TOSCANOPaul MonahanFergus W. GardinerPaul N. WhiteheadFerhat Ozgur CatakPaul S. AmieuxFermin García-Muñoz RodrigoPaul SchoenhagenFermin TorranoPaul T. ThevenotFernanda M. SilvaPaula BoaventuraFernando AlmeidaPaula Fernández GarcíaFernando FerreiraPaula Fernández-PiresFernando Jesús Plaza Del PinoPaula PeixeFernando Leiva-CepasPaula Peral GómezFernando Martín-RiveraPaula ProcterFernando RiegelPaulina WignerFernando SotoPaulo AcioliFernando Suparregui DiasPaulo LopezFilipe BarrosoPaulo MeloFilipe Manuel ClementePavan MadanFilippe Falcao-TebasPavel AvdoninFilippo GiordanoPaweł ChruścielFilippo MacalusoPaweł DymoraFilippo MaselliPawel LinekFilippo RebessiPawel MacekFiona GardnerPearl ChenFiorella GuadagniPedram P. SendiFiras KobeissyPedro A. Martin CervantesFlávia Aparecida GraçaPedro AlbarránFlávia Venetucci GouveiaPedro Bertemes-FilhoFlavio BertiniPedro De BruyckereFlavio RizzolioPedro EncarnaçãoFlora GattiPedro Fernandes AnunciaçãoFlorence Mei Fung WongPedro ForteFlorian PiekarskiPedro Henrique Lopes SilvaFlorian SchmitzPedro José Tauler RieraFlorica MoldoveanuPedro L. Pancorbo HidalgoFloris S Van Den BrinkPedro LucasFolashade Tawakalitu AllohPedro Luiz RamosFranca NatalePedro Manuel Moreno-MarcosFrances M. LesliePedro Manuel Rodríguez-MuñozFrancesca CostanzaPedro Miguel Alves Ribeiro CorreiaFrancesca DiolaiutiPedro Miguel CarvalhoFrancesca ErvasPedro Miguel FariaFrancesca RizzelloPedro Miguel SardoFrancesca RossiPedro MorouçoFrancesca ZottiPedro Plans-RubióFrancesco AgostiniPenelope W. St. J. WatsonFrancesco BaccelliPennie J TaylorFrancesco BennardoPercy NohamaFrancesco BiancoPerman GochyyevFrancesco CantielloPeta MurrayFrancesco CauteruccioPeter BesharaFrancesco CiodaroPeter Bob PeerenboomFrancesco CoreaPeter EibichFrancesco De MiccoPeter GänglerFrancesco Di GennaroPeter J. AspinallFrancesco MantaPeter KaatschFrancesco Maria GalassiPeter Kenneth HuggardFrancesco SalustriPeter KernFrancesco TramaPeter MurrayFrancesco Vincenzo SambatiPeter SchmidtFrancesco ZarantonelloPeter V. SmeetsFrancisca López TorrecillasPeter W. ChoateFrancisco AlenPeter WakholiFrancisco Caamaño IsornaPeter-Michael SackFrancisco CercasPetr KudrnaFrancisco Flor-MontalvoPetra KarlssonFrancisco Garrido PeñaPetra PoulováFrancisco Guillen-GrimaPetri BöckermanFrancisco IacobelliPetrona LeeFrancisco J. MedinaPhilip LiFrancisco Javier Cano-GarcíaPhilip TsengFrancisco Javier Fernández CarrascoPhilip W. WertzFrancisco José Rodríguez VelascoPhilippe GuillaumeFrancisco Luna-PerejónPhilippe J. GiabbanelliFrancisco Manuel Melgarejo MeseguerPhyllis AnastasioFrancisco MartinPhyllis GasparFrancisco Miguel Escandell-RicoPhyllis GendlerFrancisco Pablo Holgado-TelloPierfrancesco FrancoFrancisco Pedro García-FernándezPiergiorgio IannoneFrancisco PerezPierre BoulangerFrancisco RiveraPierre ChausséFrancisco RodríguezPietro CiampiFrancisco Salguero-CaparrósPietro CinagliaFrancisco SampaioPietro FerraraFranco BagnoliPietro MontemezziFranco BassettoPietro PicernoFrançois GravellePilar Alfageme-GarcíaFrançois MarguerittePilar Aparicio-MartinezFrans VisserPilar CarreraFraser CarsonPilar Marques-SanchezFred MasonPilar Pérez-RosFrederic DenisPinakin DaveyFrederic SchoenbergPinchas HalperinFredericus H. J. Van LoonPing WangFrederik PetersPiotr DługoszFrederike T. FellendorfPiotr DobrowolskiFredrik MolinPiotr GawdaFredrik VelanderPiotr KornetaFrida JonssonPiotr LeszczyńskiFrutuoso G. M. SilvaPiotr PoznańskiFuhmei WangPiyush RanjanGabriel Fuertes MuñozPo-Chang HsuGabriela TopaPoppy NicolGabriele CapursoPouya HassandarvishGabriele CentiniPouya JavadianGabriele CervinoPradeep PodilaGabriele MascheriniPradeep VaitlaGabriele PesimenaPrajakta AdsulGabriele PiuriPranshu SahgalGabriele SavioliPrasad NalabothuGabriele TonniPrasenjit SahaGabriella CasalinoPrashilla SomaGabriella PravettoniPrateek LohiaGaetano GalloPritam BoseGaetano PaolonePriyanka AgarwalGagan KumarPriyanka KhareGaia SampognaProf. Leonie ShortGalateja JordakievaProfeta MartinaGang-Hoon SeoPrzemyslaw Falkowski-GilskiGarasic Mirko DanielPrzemysław LisińskiGarcía-Moreno Nisa FranciscaPuppo FrancescoGarry KuanPuvanalingam AyyaduraiGary D. NovackP.V. RavindraGautam SrivastavaQi ShaoGazanfar RahmathullaQian DuGeerten-Has TjakkesQiang FuGema BarrientosQin Xiang NgGema Sanchis-SolerQuan-Hoang VuongGeoffrey D. KahnRachel J. SkowGeorg BolligRachel TaylorGeorge A. GellertRachele LamioniGeorge AntonogeorgosRadhi Al-MabukGeorge LatsiosRadu CiorapGeorge LazaroiuRaf L.J. MeesenGeorge LiuRafael Almendra-PeguerosGeorge MaitiRafael Gómez-GalánGeorge SilberschatzRafael López-CorderoGeorge UmemotoRafael Molina-LuqueGeorgia CasanovaRafael Vila-CandelGeorgia GomatouRafał Białynicki-BirulaGeorgia HalkettRaffaele De CaroGeorgia KaidonisRaffaele La RussaGeorgia RowleyRaffaele OrnelloGeorgios D. KotzalidisRaffaele SerraGeorgios TagarakisRahul MukherjeeGeorgiy KorobeynikovRajan SharmaGerald AranoffRajan VediappanGerald ChiRajasekhara NerimetlaGeraldine TorrisiRajeev DwivediGerhard LeitnerRaji P. GrewalGert-Jan Van Der PuttenRam BajpaiGgaby JudahRam SharmaGiacomo BaimaRamcés Falfán-ValenciaGiacomo NalliRami DiabGiacomo OteriRami S. KantarGiampiera BulfoneRamjee BhandariGiampietro FarronatoRamón FuentesGian Luca MarellaRamon GoingsGiancarlo Antonio FerroRamón González-CamarenaGianCarlo PecorariRamón Montes-RodríguezGianina LucaRamona BongelliGianluca CiardiRamos-Petersen LauraGianluca EspositoRamune JacobsenGianluca GennarelliRandall S. DaviesGianluca R. DamianiRaouf Senhadji-NavarroGianluca RizzoRaphael AraujoGianluca SerafiniRaphael R. BrunoGiannis ArnaoutisRaquel AlarcónGilberto Gonzalez-ParraRaquel BarbosaGilles LesieurRaquel García RevillaGinevra GraviliRaquel Lara MorenoGinny WebbRaquel Leirós-RodríguezGiordano AlessandraRaquel Lozano BlascoGiorgina Barbara PiccoliRaquel Vaquero-CristobalGiorgio FalgaresRasa MladenovicGiorgio LombardoRathinasamy BaskaranGiorgio MatteiRatnadeep MukherjeeGiorgio PolettiRaul DomínguezGiorgio TregliaRaúl DomínguezGiorgos K. SakkasRaúl EspertGiovanna BertiniRaúl Fernández-TorresGiovanna RicciRaúl Ferrer PeñaGiovanni BarassiRaúl Juárez-VelaGiovanni BocciaRaul Justo SanzGiovanni CiliaRaul NavarroGiovanni Codacci-PisanelliRaúl Quevedo-BlascoGiovanni Di DomenicoRaul RamosGiovanni FalsiniRaúl Soto-CámaraGiovanni IolasconRavi KudesiaGiovanni MarascoRavindra KumarGiovanni MergoniRaymond J.G. OpdenakkerGiovanni RollaRazvan SocolovGiovanni SchmidRebeca PardoGiovanni TarantinoRebecca DiekmannGiovanni ZoccaliRebecca ShanklandGirdhari RijalRebecca TsaiGirish UpretiRegis BobeGisela Redondo-SamaReima SuomiGisela Ruiz VegaReinaldo B. BestettiGisoo ShinReinhard KoppGiulia AbateReinhold FeldmannGiulia LausiRejina ManandharGiulia MoreoRemedios Lopez-LiriaGiulia PurpuraRena HiraniGiulia RodariRenata Lopes RosaGiulio Di GravioRenata Puppin ZandonadiGiulio DiMizioRenbin XiaoGiulio PirottaRene KadenGiulio PretiReneta BarnevaGiuseppe ComentaleRen-Jei ChungGiuseppe DionisioReuben HowdenGiuseppe FiorentinoReza AlaviGiuseppe ForteReza SaatchiGiuseppe GiudiceRezzy Eko CarakaGiuseppe LanzaRhonda MattinglyGiuseppe LiscoRiasat HasanGiuseppe MusumeciRičardas RadišauskasGiuseppe NassoRicardo AlmendraGiuseppe OrlandoRicardo BrownGiuseppe PonziniRicardo Fueyo-DíazGiuseppe PortellaRicardo Javier Cerda RiosecoGiuseppe RivaRicardo Jorge Dinis-OliveiraGiuseppe SammarcoRicardo Martino AlbaGiuseppe SantarpinoRicardo Sánchez De MadariagaGiuseppina InusoRicardo Ventura BaútoGlenn FinauRiccardo BientinesiGoncalo MarquesRichard FergusonGonçalo MarquesRichard H. Parrish IIGonçalo SantinhaRichard HolyGonzalo HaroRichard MorehouseGopi K. PenmetsaRichard ParrishGopinath KrishnasamyRifat AhmedGordon HarperRiitta TurjamaaGourav BanerjeeRiki TeslerGovind KrishnamoorthyRita DeeringGowtham K AnnarapuRita Maria Vittoria NobiliGrasiele SausenRita PavasiniGrażyna BartkowiakRita Payan-CarreiraGregor StavrouRita Rahoi-GilchrestGregory ConnersRittika ShamsuddinGregory PetersonRize JingGregory SimonsRobbert GobbensGrzegorz BulajRobert A. HahnGrzegorz IlewiczRobert B. RuckerGrzegorz NowickiRobert BoydGrzegorz OnikRobert DonovanGrzegorz StarobratRobert E. PykeGrzegorz TrybekRobert HoerrGrzegorz ZielińskiRobert KellyGu Eon KangRobert M. SiegelGuan WangRobert MitchellGuang YangRobert SusloGuangli LiRobert WalinderGuan-Zheng LiuRoberta ForlanoGuennadi SaikoRoberta TaralloGuglielmo Actis DatoRoberto BenoniGuglielmo StabileRoberto CannataroGuida Da PonteRoberto Carlos Castrejón-PérezGuido BruinsmaRoberto CasconeGuido Franco AmorettiRoberto CodellaGuido VielRoberto Miniero MiieroGuillermo A. Cañadas-De La FuenteRoberto Nuño-SolinísGuillermo Cámara-ChávezRoberto ScendoniGuillermo LaheraRoberto Soto-VarelaGuillermo PetzoldRobin HealyGuillermo PlazaRocca GabrieleGundu RaoRocco ServidioGuru Raghavendra ValicherlaRocío De Diego CorderoGustavo FernandesRocio Palomo-CarriónGustavo GlusmanRod MooreGustavo PimentelRoddy HiramGustavo R MotaRodney P JonesGuy TraininRodrig MarculescuGwenaël PiaserRodrigo España-NavarroHadeel HalawehRodrigo Poblete UmanzorHaewon ByeonRodrigo SalasHae-Young KimRodrigo Torres-CastroHaichao WeiRodrigo ValenzuelaHai-Hua ChuangRoel Van OvermeireHaishu LinRoger HoHaitham NobaneeRoger WatsonHamid Alinejad-RoknyRohail HassanHamid JafarzadehRohan NagareHamid SaadatfarRohan SharmaHang Nga MaiRoma JusienėHang XingRoman Fouad MansorHania Salah ZayedRoman KrálikHanifa BachouRomana SchirhaglHanna GerberRomany F. MansourHan-Na KimRomina FucàHanna-Kaarina JuppiRonald B. BrownHanns de la Fuente-MellaRonald HinchHans HobbelenRondy YuHans-Christian KolbergRong LiuHany HassaninRoopma WadhwaHanyong PengRosa Carla SilvaHan-Yu LinRosa Del CampoHaobijam BasantaRosa Magallón-BotayaHarald StummerRosa Maria Tapia-HaroHarikumar RajaguruRosanne A. CouttsHarish GargRosaria RuccoHarland HolmanRosaura LeisHarold MourasRose BakerHarry H.X. WangRosella CataldoHartmut F. WitteRosemary CaronHasmun NorenRosie NelsonHassan DoostiRosita RupaHayato TsukamotoRossella MarmoHe ShuhanRoswith RothHéctor Beltrán-AlacreuRoxana StegaroiuHéctor García- LópezRuaraidh DobsonHee-Taik KangRubén Abraham Domínguez-PérezHeewon JungRuben Blachman-BraunHeidi HillmanRubén Francisco González-LaredoHeidi TuronRuben MedinaHeike Maria ElfleinRubén Sánchez-GómezHeike RudolphRüdiger PryssHeike WieserRudolf LikarHeiko BrennenstuhlRui CamachoHeiko TzschatzschRui LiHeinrich WeberRui NevesHei-sung KimRui PoinhosHelbert Eduardo EspitiaRui SilvaHelen AlmondRui XuHelen McaneneyRuifeng HuHelena BergströmRuixue HouHelena CatarinoRuoxi WangHelena Fernández LópezRuslan DorfmanHelena JoséRussell D'souzaHelge L. WaldumRut Lucas-DomínguezHelge StalsbergRuxandra Sava-RosianuHelmut FrohnhofenRyoma MichishitaHelmuts AzacisRyota ShizuHelvio AffonsoRyuichi OhtaHema KesaS. Cristina OanceaHenin DolajiSaaima HasninHenk KoppelaarSaba TabasumHenri TilgaSabina Barrios-FernándezHenriette HeinrichSabina La GruttaHenriëtte PrastSabina WagnerHenrique CastroSabine LemoyneHenrique NascimentoSabine LichteneggerHenrique PereiraSabrina BonichiniHenry XiangSachiko OzoneHerbert ChikafuSachit AnandHerbert Ryan MariniSaeed MohammadiHerrera-Guerra AngelSaeed UL HassanHervé MénardSaeid Abbasi-MalekiHidenori KomiyamaSaeideh Valizadeh-HaghiHidenori SuzukiSafeera HussainyHideo KatoSagrario Gomez-CantarinoHimanshi AggarwalSagrario Pérez- De La CruzHimanshu DeshwalSahar HoushdaranHirayuki EnomotoSaïd AssarHiro KishimotoSai-fu FungHiroki TeragawaSaifudeen IsmaelHiroko MiuraSajal K. SahaHiroko OchiaiSakari B. SuominenHiromichi ItoSakeenabi BashaHironori IshiguchiSaket A KottewarHiroshi IrisawaSakiko KanbaraHiroshi KatagiriSally GuttmacherHiroshi KurokiSally NashHirotada MoriSalvador Angosto SánchezHiroyoshi Y. TanakaSalvatore GiordanoHiroyuki FukuiSalvatore Giovanni VitaleHiroyuki NakamuraSalvatore LopreviteHiroyuki SuzukiSalvatore ZaffinaHisanori FukunagaSamaneh MadanianHo Ting WongSamar HelouHo TsoiSambit MishraHon Lon TamSamir MalakarHongming ShanSamue AtchersonHongsun GuoSamuel Fernandez SalineroHongying PanSamuele CerutiHon-Yi ShiSamy M. RiadHossein ZareSandesh PanthaHotcherl JeongSandhiran PatchayHoward SelingerSándor BenyheHsiao-Mei ChenSandra AlhoyekHsin-Chen LeeSandra ChistoliniHsin-Chieh LanSandra Montagud-RomeroHsiu-Ching ChiuSandra SchüsslerHsu-Heng YenSandra V. FernandezHu DingSandro Arrufat-MartínHu LiuSandro RengoHugo CardosoSandro SerpaHugues LamotheSandy J. LwiHui HuangSang Suk KimHuijun ChihSang‐Dol KimHumberto CarvalhoSang-gyung KimHung-Pin TuSang-Ho KimHung-Yi ChuangSanjeevan MuruganandanHyechang RhimSanket MishraHye-geum KimSanne F.E. RoversHyoung Suk LeeSantiago Giraldo LuqueHyoung Woo ChangSantiago Martínez-IsasiHyoung-Kil KangSantiago MoraHyungju KwonSara B. DonevantHyun-Hee HeoSara BernardiHyun-jin MinSara CondinoHyunsoo LeeSara MocciaHyun-sun ParkSara MonteiroIan CumminsSara PerettiIbolya FülöpSarah CatlowIbraheem M. KarayeSarah DavenportIbrahem KandelSarah FriedrichIbrahim HassanSarah HodgeIchiro YamadaSarah J. AllgoodIcíar ArteagoitiaSarah LehmanIdiano D'AdamoSarah M. CamhiIe-Bin LianSarah M. Dos AnjosIffath Unissa SyedSarah ManessIgnacio Díez LópezSarah PriorIgnacio Martínez-González-MoroSarah SnuggsIgnacio Ramos-VidalSarah VordenbergIgor GrabovacSatarupa DasguptaIgor GruićSatinder DahiyaIgor NestrasilSatoshi KashiwagiIkuo InoueSatoshi OsukaIlaria CampioniSaudade LopesIlaria CataldoSaúl Martinez-HortaIlaria RiboldiSavina SchoenhoferIlaria RubinoSayyed Fawad Ali ShahIlias DoulamisSchauer MatthiasIlmari Määt­tä­nenScott D. GrosseIlyong JiScott M. DebbIm SunScozzari GitanaImmacolata Cristina NettoreSebastian KrapohlIndika KahandaSebastiano FaroIndu G. RajapakshaSebastiano MassaroInês BaíaSebastiano MercadanteInes Bilic-ĆurčićSefika KiziltoprakInés Casado-VerdejoSegundo Nicolas SeclenInes DespotovicSeigo OhbaInes TestoniSeka LazareInga SaitadzeSelcuk KucukseymenInge WernerSelene BaroneIngela MarklinderSema K. AydedeIngrid TonniSeok-Ki JungIn-Jeong KimSeong Man ParkInmaculada Concepción Rodríguez-RojoSeongil LeeInmaculada García-MartínezSeong-Kyu KangInmaculada Mateo-RodríguezSerafeim C. ChaintoutisInsil JangSerena DattolaIoana Dana AlexaSerge BrandIoanna MylonaSergey MaksimovIoannis PaliokasSergi Garcia-RetortilloIoannis PanagisSergio A. SilverioIoannis VamvakarisSergio A. UsecheIonela Ruxandra SfeatcuSergio Cinza-SanjurjoIonut LuchianSergio López-GarcíaIosif SifakakisSerkan VarolIosune Salinas-BuenoSeul-Ki LeeIranzu Mugueta-AguinagaSeung-ha LeeIrena IlicSeunghoo LimIrena TušerSeungwon KwonIrene AgeaSe-Youn JungIrene AlbarránShabnam Jalili-MoghaddamIrene CeccatoShahab HaghayeghIrene KamenidouShamik ShahIrene Torres-SanchezShamika AlmeidaIreneu MendesShandell ElmerIrina MocanuShane L. RogersIrina St. LouisShanina KnightonIrit KeynanShannon FarmerIsaac Machorro-CanoShanquan ChenIsabel Corrales-GutiérrezShaohua WanIsabel Del ArcoSharda KumariIsabel FernandesSharon DavideskoIsabel LucasSharon L. LarsonIsabel María Fernández MedinaSharon RennerIsabelle SkinnerShayne Baker OAMIsam BsisuSheena LeekIsdore Chola ShamputaSheila M. CampbellIshmael Festus JajaSheila Sánchez CastilloIsmail LaherShen YinIssam TanoubiSheng-Yu FanIstván BudaiShepard HurwitzIstván RuzsicsShi ChenIulia-Cristina StanicaShib Sankar SanaIván Herrera-PecoShigeyuki HamoriIvan KristoShih-bin SuIvan ManziniShihoko Komine-AizawaIvan MiskulinShijie ZhouIvan RuchkinShikui WuIvana KholováShin Jun ParkIvana SutejShingo YamamotoIvo DraganovShinichi YoshiokaIvonne NelShinji TeramotoJ. Grant McFadyenShinta NishiokaJ. JumadiShintaro FujiharaJacek BarcikShintaro SengokuJacek BoguckiShin-Tsu ChangJacek WoźniakShiro MizunoJacinto JardimShreoshi Pal ChoudhuriJack ThepsourinthoneShuangge MaJackline Freitas Brilhante De Sao JoseShuichi SatoJacob GrazerShumit SahaJacobo Rodríguez-SanzShunsuke KurodaJacopo SabbatinelliShu-Zhi LiuJacqueline E. ReznikShyam Kumar GudeyJacques CabaretSiamak MoayediJacques PendersSiddharth JaggavarapuJade Lai King WongSigne TomsoneJadwiga AmbroszkiewiczSijian LiJae Bum ParkSilvana Nair LeiteJaehee LeeSilvano DragonieriJaeho ChoSilvia de Miguel-BilbaoJae-Hong KimSilvia García-BujalanceJae-Ik ShinSilvia MarcuJaejong ParkSilvia MonacoJaime Martinez-CastilloSilvia Portero de la CruzJames BrasicSilvia Regina Dias Medici SaldivaJames EdwardsSilvia SalvatoreJames G. RavinSilvia SoleJames L. VadenSimon Bheki KhozaJames PethickSimon PorcherJames PowersSimona Cătălina ŞtefanJames SonneSimona Di FrancescoJames W. SonneSimona MandiniJan A.P.M. De LaatSimone BelliJan BilskiSimone Domenico AsprielloJan DomaradzkiSimone VarrasiJan DommerholtSina HafiziJanaina Antonino PintoSiobhan AaronJanani IyerSiqin WangJane E. KrauseSisitha JayasingheJanek MusekSławomir DreslerJanelle SkinnerSlawomir JarekJanet DafoeSławomir PalickiJanet MattssonSławomir WołczyńskiJang-Hee ChoSofi FristedtJangwon GimSohei SatoiJannick NiortSomayeh PouyanfardJansen N. SeheultSong WangJanusz MyszczyszynSongee JungJanusz SielskiSonia GonzálezJanusz SobeckiSonia HornJanusz WielkiSonia J. Romero MartínezJarim KimSonia LippkeJarosław HerbertSonia M. OliveiraJaskaran Singh SethiSonia UdodJason BrumittSonia ValJason CooperSonja BidmonJason PoleSonja J EllisJason S. BuhrmanSonu Menachem Maimonides BhaskarJason W. MarionSoo-Hwan ByunJasper VerheulSoo-Hyun SungJavier A. Arrieta-EscobarSophia BoudjemaJavier C. AnguloSophie CarterJavier CarniceroSophie RutterJavier E. Contreras-ReyesSøren MikkelsenJavier J. PerezSorin HostiucJavier LopezSou Hyun JangJavier Ortuño SierraSouhail HermassiJavier P. GisbertSoundappan S.V. SoundappanJavier Rodríguez VillanuevaSrujana SahebjadaJavier SantabarbaraStefan BratosinJay MichaelsStefan SeifertJay MishraStefano D'ErricoJayakrishnan AjayakumarStephanie DuguezJayce FarmerStephanie Y. CrawfordJ.E. Trinidad-SegoviaStephen E. ForemanJean AmiralStephen Siu-Yu LauJean Claude De MauroySteve GlassJean Michel MaixentSteven BaumruckerJean WooStuart J. OlshanskyJean-Christophe GigerStylianos MystakidisJean-Jacques MonsuezSudip MondalJean-Pierre JesselSudipta BiswasJean-Pierre MichelSu-Lien LuJeconiah Louis DreisbachSultan Ayaz AlkayaJeff GibbonsSung-Hyoun ChoJeff HatalaSun-Joo JangJeff SandsSunny RajJeffrey C. F. HoSusan BoonJelena BobkinaSusan RamloJelena RoganovicSusan RifkinJeng-Shyang PanSusana García-SilvaJennifer BraySusana Llorens GumbauJennifer ChippsSusana RubioJennifer CreeseSusana Rubio-ValdehitaJennifer DanielsSusumu ItoJennifer GatzemeierSuzana OtaševićJennifer GrossmanSvetlana SarantsevaJennifer HowcroftSyed Ehtisham-ul-HaqueJennifer K. YeeSygkliti-Henrietta PelidouJennifer L. ScheidSylwia ChojnowskaJennifer M. WhittingtonSzabo Dan-AlexandruJennifer XieTa-Chuan YehJenny DrottTadashi ItoJenny QueTae Im KimJenny WilkinsonTae Keun YooJens Christoffer SkogenTaewoo NamJens KirchnerTakahiro KuboJeonghoon HaTakamasa MizunoJeong-won HanTakamichi MorikawaJer-Ming ChangTakashi IshigeJerónimo GonzálezTakashi MatsudaJerry PullanTakashi SatoJerzy ChudekTakayuki NittaJerzy GębskiTakemi OtsukiJesse MathesonTao HuangJessica García-GonzálezTapas RayJessica GuerraTasuku OkuiJessica HardtTatiana Viktorovna KochetovaJesuk KoTeruyoshi AmagaiJesús IbarluzeaThar BakerJesús MartínezTheodora PapamitsouJesús Molina-MulaTheodoros GiannouchosJesús Rivera-NavarroThi Hong TranJesús-Nicasio García-SánchezThierry Oscar EdohJeungchan LeeThomas DandekarJi Hwan ParkThomas PlatzJi Rak KimThomas WenzelJiacun WangThorpe RachelJian GaoTianjun WangJian WengTomasz BlicharskiJianfeng LiTomasz M. KarpińskiJianguo HaoTomoki NakamizoJianping MaTomoko ItoJianye LiuTomoya ItataniJianzhi LinTomoyuki KuwakiJijia WangTomoyuki MukaiJill Susan SimonianTsai Chia-LiangJillian Vinall MillerTsubasa KawasakiJi-Man ParkTuyen Van DuongJin LiuUlises CortésJina OhVaitsa GiannouliJinbao GuValentina Oana BudaJin-Ding LinVanesa Cantón-HabasJing WuViacheslav OsadchyiJing YaoVicente Gea-CaballeroJingjing LuoVictor Manuel Mendoza-NuñezJingjing TangVictoria BelousovaJin-Hwa JungVincenza GianfrediJin-tae HanVincenzo De CeglieJiri KassaVinny NegiJiseun LimViraj MuthugalaJoan Francesc FondevilaVirginia RecchiaJoana Sofia Dias Pereira De SousaVladimir RogalewiczJoan-Francesc Fondevila-GascónWalter R. SchummJoanna BaranWalter Y H LamJoanna Janiszewska-OlszowskaWei-Chieh LeeJoanna MazurWeihua OuJoanna RógWeisheng ChiuJoanna SimosWesley KephartJoanna ŚliwaWilliam R. RodríguezJoanna WachnickaXiangpeng LiaoJoanna WangXiaoyan ChenJoanna WitkosXóchitl TrujilloJoanne MurrayXudong HuangJoannes ChliaoutakisXuejun DingJoão BotelhoYachao LiJoão Carlos MacedoYamakawa MiyaeJoão ClaudinoYasinta Astin SokangJoão F. C. A. MeyerYasuhiro MikiJoão Filipe Fernandes Lindo SimõesYeong Jun JuJoao MamedeYi LiuJoão Manuel Graça FradeYi-Han LuJoão Manuel Mendes CaramesYi-Hsuan HsiaoJoão MartinsYingpeng LiuJoão Paulo Mandes TribstYin-Hwa ShihJoão Pedro GregórioYirui LiangJoão SerranoYi-Wen ChiuJoaquín Cayón De Las CuevasYong Il HwangJoaquin González-FuentesYongfeng LiuJoaquín López-PascualYoshifumi ManabeJoaquín NavajasYoshihiro FukumotoJoaquin Reverter MasiàYoshiro HoraiJoël CollocYoung Sook RohJoel JamesYoung-Chang P. AraiJoelle KarlikYounjae OhJoeri Jan PenYoussef RomanJohannes PernaaYuan-Ti LeeJohannes ZipperleYuan-Yuan WangJohn A. St. CyrYu-Chao ChangJohn Amson CapitmanYu-Chi LeeJohn BartkowskiYu-Chu HuangJohn CarusoYuhei OtobeJohn CokleyYuji KabasawaJohn CoulterYuka KotozakiJohn F. NewmanYukiko OkamiJohn G. KellettYun-seob LeeJohn GunnZargari Marandi RamtinJohn H. KindredZhong GuozhangJohn J. GuersZibing JinJohn MacKayZiheng ShangguanJohn TowerZsofia LazarJolanta Klukowska-RötzlerZsuzsanna LichnerJolanta Kucharska-Mazur



